# Died

**Published:** 1892-04

**Authors:** 


### Died.

Dr. Jos. G. Cameron, in the 65th year of his age, on March 5, 1892.

The death of this worthy member of the Dental Profession of this city (Cincinnati), removes from us one of our oldest members, and the longest in practice of any now practicing in this city. He was one of the last of that group of men who did so much by their skill as expert workmen in gold and silver, by utilizing their handicraft in producing fine specimens of gold plate work to replace the lost teeth ; and he added improvements in the structure of plates by adding the beautiful rims around the plates, giving at once a finish to a gold plate that marked it as a work of skill and admired by all at our convention in days long ago. He was ever ready to appreciate and adopt everything that was valuable to the profession, evincing a warm and active interest in Dental College affairs. He was for many years a Trustee, Treasurer and Secretary of the Ohio College. He was comparatively young, dying at the age of sixty-five, his death perhaps hastened from an accident in his practice by wounding his finger with an excavator, causing blood poisoning. He recovered but the attack left him feeble, and he succumbed to a cold and pneumonia, which at last ended his long and laborious life. He enjoyed a large practice so long as he was able to attend to it, and is missed by a large clientele. He was an honest man, retiring in his disposition yet active in all matters pertaining to the profession, and was considerate and kind to every member of the profession, ever ready and pleased to give any information to younger members when desired; and we, who have known him many years, feel that we have lost a friend of the profession, a man that did much to elevate the standard in his special department of Dental Art. As a citizen he was esteemed ; and as a Christian he was beloved by the church of his choice in which he had been an officer over 30 years.

To his bereaved wife and family in their great sorrow we tender our deep sympathy ; and may the memory of his beautiful life be cherished and imitated by his sons he loved and has left.

Signed.

DR. JAMES LESLIE,

DR. J. S. CASSIDY,

DR. M. H. FLETCHER.

Committee.

